# *Anaplasma phagocytophilum* Induces TLR- and MyD88-Dependent Signaling in *In Vitro* Generated Murine Neutrophils

**DOI:** 10.3389/fcimb.2021.627630

**Published:** 2021-03-04

**Authors:** Beate J. Müller, Arne Westheider, Katharina Birkner, Birte Seelig, Susanne Kirschnek, Christian Bogdan, Friederike D. von Loewenich

**Affiliations:** ^1^Institute of Medical Microbiology and Hygiene, University of Freiburg, Freiburg, Germany; ^2^ Mikrobiologisches Institut—Klinische Mikrobiologie, Immunologie und Hygiene, Universitätsklinikum Erlangen and Friedrich-Alexander-Universität (FAU) Erlangen-Nürnberg, Erlangen, Germany; ^3^ Medical Immunology Campus Erlangen, FAU Erlangen-Nürnberg, Erlangen, Germany; ^4^Department of Medical Microbiology and Hygiene, University of Mainz, Mainz, Germany

**Keywords:** *Anaplasma phagocytophilum*, TLR, NLR, CLR, MyD88, iNOS, chemokine, cytokine

## Abstract

*Anaplasma phagocytophilum* is a tick-transmitted obligate intracellular Gram-negative bacterium that replicates in neutrophils. It elicits febrile disease in humans and in animals. In a mouse model, elimination of *A. phagocytophilum* required CD4^+^ T cells, but was independent of IFN-γ and other classical antibacterial effector mechanisms. Further, mice deficient for immune recognition and signaling *via* Toll-like receptor (TLR) 2, TLR4 or MyD88 were unimpaired in pathogen control. In contrast, animals lacking adaptor molecules of Nod-like receptors (NLR) such as RIP2 or ASC showed delayed clearance of *A. phagocytophilum*. In the present study, we investigated the contribution of further pattern recognition receptor (PRR) pathways to the control of *A. phagocytophilum in vivo*. Mice deficient for the NLR NOD2 had elevated bacterial loads in the early phase of infection, but were unimpaired in pathogen elimination. In contrast, animals lacking adaptor proteins of different C-type lectin receptors (CLR) such as DAP12, Fc-receptor γ-chain (FcRγ) and SYK controlled *A. phagocytophilum* as efficiently as wild-type mice. Further, we investigated which PRR pathways are involved in the sensing of *A. phagocytophilum* by *in vitro* generated Hoxb8 murine neutrophils. *In vitro*, recognition of *A. phagocytophilum* by murine neutrophils was dependent on TLR- and MyD88 signaling. However, it remained intact in the absence of the NLR NOD1, NOD2 and NALP3 and of the CLR adaptor molecules DAP12 and FcRγ. From these results, we conclude that TLR rather than NLR or CLR are critical for the detection of *A. phagocytophilum* by neutrophils although *in vivo* defective TLR-signaling is compensated probably because of the redundancy of the immune system.

## Introduction

*Anaplasma phagocytophilum* is a tick-transmitted obligate intracellular Gram-negative bacterium (Dumler et al., 2001). It uses the neutrophil as primary host cell (Rikihisa, 2011) and at least *in vivo*, its replication strictly depends on neutrophils (Birkner et al., 2008). *A. phagocytophilum* elicits febrile disease called granulocytic anaplasmosis in humans (Ismail and McBride, 2017) and in animals such as dogs (Carrade et al., 2009), horses (Saleem et al., 2018), cats (Lappin, 2018), sheep, cattle and goats (Atif, 2015). Clinical signs and laboratory changes are similar in humans and animals and comprise fever, inappetence, arthralgias, leukopenia, and thrombopenia (Stuen et al., 2013).

The striking tropism of *A. phagocytophilum* for neutrophils has stimulated the interest in its immunological control which was studied primarily using the laboratory mouse as model organism. C57BL/6 wild-type (WT) mice developed transient bacteremia, but no clinical signs and eliminated *A. phagocytophilum* within 14 days post infection (p.i.) (von Loewenich et al., 2004). IFN-γ-deficient mice had elevated bacterial loads in the early phase of infection, but were unimpaired in pathogen elimination (Akkoyunlu and Fikrig, 2000; Martin et al., 2000; Borjesson et al., 2002; Birkner et al., 2008). In *A. phagocytophilum* infected animals, NK cells (Birkner et al., 2008; Pedra et al., 2008), NKT cells (Pedra et al., 2008), and CD4^+^ T cells (Pedra et al., 2007a; Pedra et al., 2007b) have been described as source of IFN-γ. NK cell derived IFN-γ was probably induced by type I IFN and IL-12 (Birkner et al., 2008). IFN-γ production of CD4^+^ T cells was IL-12 and IL-18-dependent (Pedra et al., 2007a; Pedra et al., 2007b). In contrast to the early phase of infection, bacterial clearance was independent of IFN-γ, but strictly required CD4^+^ T cells (Birkner et al., 2008). In the absence of MHC class II-restricted CD4^+^ T cells, mice were persistently infected at least until day 77 p.i. (Birkner et al., 2008). However, the effector mechanism underlying the CD4^+^ T cell-dependent control of *A. phagocytophilum* still remains to be determined.

A number of *in vivo* studies analyzed the pattern recognition receptor (PRR) pathways that are triggered by *A. phagocytophilum*. In mice, the elimination of *A. phagocytophilum* was independent of Toll-like receptor (TLR) 2, TLR4 and MyD88 (von Loewenich et al., 2004; Pedra et al., 2007b), the main adaptor molecule of the TLR (Takeda and Akira, 2004). Given the apparent lack of TLR involvement *in vivo*, Nod-like receptor (NLR) pathways (Kim et al., 2016) have been investigated. Mice deficient for RIP2 (RIPK2), the adaptor molecule of the NLR NOD1 and NOD2, showed delayed clearance of *A. phagocytophilum* lasting until day 20 p.i. (Sukumaran et al., 2012). Other NLR such as NALP3 (NLRP3) and NLRC4 (IPAF) associate with the adaptor molecule ASC (Kim et al., 2016). Mice deficient for NLRC4 or ASC had elevated bacterial loads in the early phase of infection but were able to finally eliminate the pathogen (Pedra et al., 2007a). In contrast, the course of infection in NALP3^-/-^ animals did not differ significantly from WT mice (Pedra et al., 2007a). Thus, long-term persistence as in CD4^+^ T cell-deficient mice was not observed in animals lacking key components of TLR and NLR signaling.

*In vivo*, the deletion of a defined PRR might not have an overall effect on pathogen load because of compensatory activity of other PRR. However, the signaling in one particular cell type such as the neutrophil could be affected which can be studied *ex vivo* or *in vitro*. In this context, the immortalization of murine hematopoietic progenitor cells by conditional expression of estrogen-regulated Hoxb8 allowed the production of large amounts of neutrophils (Wang et al., 2006). The infection of murine *in vitro* generated Hoxb8 neutrophils with *A. phagocytophilum* induced the expression of inducible or type 2 nitric oxide synthase (iNOS or NOS2) mRNA and the secretion of significant amounts of MIP-1α (CCL3), RANTES (CCL5), and TNF (Gussmann et al., 2017). Further, bacterial growth was significantly impaired in IFN-γ-stimulated Hoxb8 neutrophils (Gussmann et al., 2017). Thus, neutrophils seem to recognize *A. phagocytophilum* and to contribute to its control.

In the present study, we first investigated *in vivo* the role of further PRR pathways including signaling *via* C-type lectin receptors (CLR) (Brown et al., 2018) for the control of *A. phagocytophilum*. Secondly, we analyzed *in vitro* the ability of Hoxb8 murine neutrophils with defect for different PRR pathways to sense *A. phagocytophilum*. We found here that NOD2^-/-^ mice had elevated bacterial loads in the early phase of infection, but were unimpaired in pathogen elimination. In contrast, animals lacking the CLR adaptor proteins DAP12, Fc-receptor γ-chain (FcRγ), or SYK controlled *A. phagocytophilum* as efficiently as WT mice. *In vitro*, we observed that *A. phagocytophilum* might be recognized by neutrophils in a TLR- and MyD88-dependent manner. However, the NLR NOD1, NOD2 and NALP3 and the CLR adaptor proteins DAP12 and FcRγ were not involved in sensing of *A. phagocytophilum* by neutrophils.

## Material and Methods

### Mice

C57BL/6N WT mice were purchased from Charles River Laboratories (Sulzfeld, Germany). Breeding pairs of gene-deficient mice were obtained from the following sources. C57BL/6J TCRb^-/-^ TCRd^-/-^ (stock number 002122) and C57BL/6J NOD2^-/-^ mice (stock number 005763) were bought from the Jackson Laboratories (Bar Harbor, ME, USA). C57BL/6J TRIF^Lps2/Lps2^ mice were provided by Peter Staeheli (University of Freiburg, Freiburg, Germany). C57BL/6J DAP12^-/-^ and C57BL/6J NOD1^-/-^ mice were obtained from Andreas Diefenbach (Charité, Berlin, Germany). C57BL/6J FcRγ^-/-^ mice were provided by Hanspeter Pircher (University of Freiburg, Freiburg) and C57BL/6J SYK^flox/flox^ mice by Robert Zeiser (University of Freiburg, Freiburg, Germany). C57BL/6J LysM^cre/cre^ mice were obtained from Attila Mócsai (Semmelweis University, Budapest, Hungary). To generate mice with SYK-deficient myeloid cells C57BL/6J SYK^flox/flox^ and C57BL/6J LysM^cre/cre^ mice were crossed. C57BL/6J mice defective for TLR 2, 3, 4, 7, 9 (TLR-5-fold) were provided by Carsten Kirschning (University of Duisburg-Essen, Essen, Germany). C57BL/6J MyD88^-/-^, C57BL/6 TRIF^-/-^ and C57BL/6 TLR9^-/-^ mice were obtained from Hermann Wagner (Technical University of Munich, Munich, Germany). C57BL/6J TLR7^-^/^-^ were provided by Stefan Bauer (University of Marburg, Marburg, Germany). C57BL/6J NALP3^-/-^ were obtained from Uwe Ködel (Ludwig-Maximilians-University, Munich, Germany). All mice were housed under specific pathogen-free conditions and used at the age of 6–12 weeks. The animal experiments were approved by the animal welfare committee of the Regierungspräsidium Freiburg (G-06/19 and G-11/79).

### *In Vivo* Infection Experiments

The *A. phagocytophilum* Webster strain (Asanovich et al., 1997) was maintained through continuous passage in infected C57BL/6 TCRb^-/-^ TCRd^-/-^ mice and used for mouse infection experiments as reported previously (von Loewenich et al., 2004). In general, groups of three mice were sacrificed at different time points after infection. EDTA-anticoagulated blood, spleen and lung were collected from each animal. Samples were subjected individually to DNA preparation and quantitative PCR analysis as reported previously (von Loewenich et al., 2004). The bacterial load was calculated as copies *A. phagocytophilum*/copy murine glucose-6-phosphate dehydrogenase × 10^−3^.

### Hoxb8 Neutrophils

Female mice were used at the age of 8–12 weeks. Progenitor cells were derived from murine bone marrow. The progenitor cells were retrovirally transduced with estrogen-regulated Hoxb8 and selected for 4 weeks in the presence of stem cell factor (SCF) to generate neutrophil progenitor cell lines (Wang et al., 2006). Polyclonal progenitor cell lines were cultured in Opti-MEM + GlutaMAX medium (Life Technologies, Darmstadt, Germany) supplemented with 10% FCS, 30 µM ß-mercaptoethanol, 1 µM ß-estradiol (Sigma-Aldrich, Taufkirchen, Germany) and 1% supernatant from SCF-producing CHO cells. The SCF producing cell line was kindly provided by Hans Häcker (St. Jude Children’s Research Hospital, Memphis, TN, USA). Differentiation was induced by ß-estradiol removal. The differentiation status of the cells was controlled for each experiment microscopically by Giemsa staining and was similar for WT and gene-deficient Hoxb8 neutrophils. MyD88^-/-^ TRIF^-/-^ Hoxb8 neutrophils were a gift from Hans Häcker.

### *In Vitro* Experiments

The *A. phagocytophilum* Webster strain (Asanovich et al., 1997) was routinely grown in differentiated Hoxb8 neutrophils and was passaged every 3 to 4 days. To determine the percentage of infected cells, cells were cyto-centrifuged onto glass slides using a Cytospin 4 centrifuge (ThermoFisher Scientific, Langenselbold, Germany) and stained by Diff-Quick (Dade Behring, Marburg, Germany). 200 cells were counted at 1,000-fold magnification. Host-cell free *A. phagocytophilum* obtained from 3 x 10^7^ Hoxb8 neutrophils with an infection rate of 90% was used to infect 1.2 x 10^7^ Hoxb8 neutrophils (differentiated for 4 days) in 6 ml medium. To separate *A. phagocytophilum* from its host cells, the infected Hoxb8 neutrophils were passaged 10 x through a 27 G needle. Subsequently, a differential centrifugation step (10 min 750 g, 10 min 2,300 g) was performed and the pellet used for the infection. Pellets prepared from 3 x 10^7^ uninfected Hoxb8 neutrophils served as control stimuli. At the time points 0, 24, 48, 72, and 96 h 500 µl from each set of samples were collected. The pellet was re-suspended in RNAlater (Life Technologies) and stored together with the supernatant at -80° C. Depending on the experiment, cells were stimulated with 200 ng/ml *Escherichia coli* K12 D31m4 (Re) LPS (List Biologicals, Campbell, CA, USA), 5 µg/ml imiquimod (InvivoGen, Toulouse, France), 5 µM ODN 1826 (InvivoGen), 10 µg/ml iE-DAP (InvivoGen), 10 µg/ml MDP (InvivoGen), or 100 µg/ml HKMT (InvivoGen). Total RNA was prepared using TRIzol (Life Technologies), treated with TURBO DNase (Life Technologies) and reverse transcribed with the High Capacity cDNA Reverse Transcription Kit (Life Technologies). Quantitative PCR was performed on an ABI Prism 7900HT Sequence Detector (Life Technologies) using TaqMan Gene Expression Master Mix (Life Technologies) and the following assays: iNOS (Mm00440485_m1) and HPRT (Mm00446968_m1). To follow the growth of *A. phagocytophilum* in Hoxb8 neutrophils, the bacterial RNA was quantified using primers 16S RTf2 (GAG AGT TTG ATC CTG GCT CAG AA) and 16S RTr (GCT ATA AAG AAT AAT CCG TTC GAC TTG) and the 16S RT probe (Fam-ACG CTG GCG GCA AGC TTA ACA CAT-BHQ1). Respective mRNA amounts were normalized to murine hypoxanthine guanine phosphoribosyl transferase 1 (HPRT) levels. Relative mRNA expression was calculated using the ΔΔC_t_-method. Levels of murine MIP-1α (CCL3), RANTES (CCL5), TNF, IL-6, IL-1β, and KC (CXCL1) were measured in the supernatants using CBA Flex Sets (BD Biosciences, Heidelberg, Germany) and a BD LSRFortessa instrument (BD Biosciences). The analysis was performed applying the FCAP array software (BD Biosciences).

### Statistical Analysis

Differences between experimental groups were analyzed using the two-tailed Mann–Whitney test. Calculations were done by GraphPad Prism 6.07. A *p* value <0.05 was considered significant. A correction for multiple testing was not done.

## Results

### Innate Immune Recognition of *A. phagocytophilum In Vivo*

**Figure 1:**
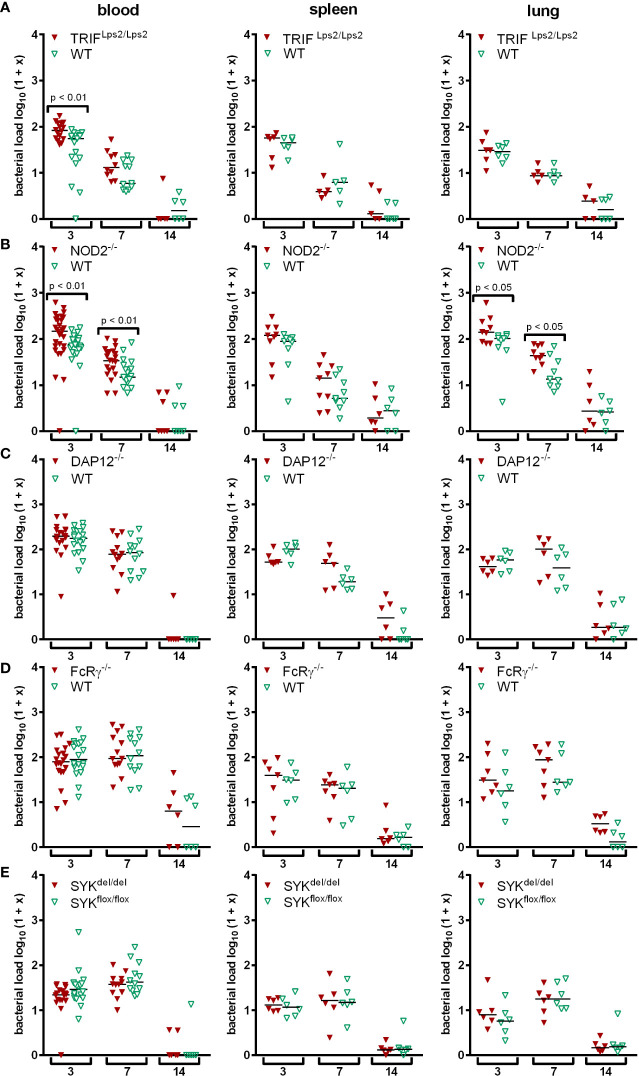
Mice were infected i.p. with *A. phagocytophilum*. Blood, spleen and lung were collected at the indicated day p.i. and their bacterial load was measured by quantitative PCR. Each data point stands for one individual mouse. The bars represent the median. Data from 2 **(A, C, D, E)** and 3 **(B)** independent experiments are shown, respectively. Differences between gene-deficient and control mice were analyzed using the two-tailed Mann-Whitney test.

*In vivo*, signaling *via* MyD88 was not crucial for the elimination of *A. phagocytophilum* (von Loewenich et al., 2004; Pedra et al., 2007b). We therefore investigated whether MyD88-independent TLR-signaling *via* the TRIF pathway (Ullah et al., 2016) could be involved. C57BL/6 TRIF^Lps2/Lps2^ (Hoebe et al., 2003) and respective WT mice were infected with *A. phagocytophilum*. Bacterial loads were determined in blood, spleen and lung at days 3, 7, and 14 p.i. Statistically significant differences between the groups were not found except for day 3 p.i. in blood (*p* < 0.01) (**Figure 1A**). This indicates that TLR-signaling *via* TRIF is not essential for the control of *A. phagocytophilum in vivo*.

Mice defective for the intracellular NLR NOD2 (Mukherjee et al., 2019) and for adaptor proteins of different CLR such as DAP12, FcRγ and SYK (Brown et al., 2018) were used to study non-TLR PRR pathways. Mice with SYK-deficient myeloid cells were generated by crossing C57BL/6 SYK^flox/flox^ and C57BL/6 LysM^cre/cre^ mice because the constitutive deletion of SYK is lethal in mice (Cheng et al., 1995; Turner et al., 1995). NOD2^-/-^ mice had elevated bacterial loads compared to WT mice in blood (*p* < 0.01) and lung (*p* < 0.05) at days 3 and 7 p.i., but were unimpaired in pathogen elimination (**Figure 1B**). In contrast, DAP12^-/-^ mice (**Figure 1C**), FcRγ^-/-^ mice (**Figure 1D**) and mice with myeloid cells lacking SYK (**Figure 1E**) were able to control *A. phagocytophilum* as efficiently as WT mice at all time points investigated. This indicates that signaling *via* NOD2 might be involved in immune recognition of *A. phagocytophilum in vivo*, whereas DAP12, FcRγ, and SYK are not.

### TLR-Dependent Recognition of *A. phagocytophilum* in Neutrophils *In Vitro*

Murine *in vitro* generated Hoxb8 neutrophils recognized *A. phagocytophilum* and secreted chemokines and cytokines upon infection (Gussmann et al., 2017). We therefore analyzed whether the absence of certain PRR pathways influenced stimulation of neutrophils by *A. phagocytophilum* and intracellular growth of the pathogen. *In vitro* generated murine Hoxb8 neutrophils (Wang et al., 2006) defective for MyD88, MyD88 + TRIF, TRIF, TLR 2, 3, 4, 7, 9 (TLR-5-fold) were used first.

#### Growth of *A. phagocytophilum* in TLR-5-fold^-/-^, MyD88^-/-,^ and MyD88^-/-^ TRIF^-/-^ Hoxb8 Neutrophils

**Figure 2:**
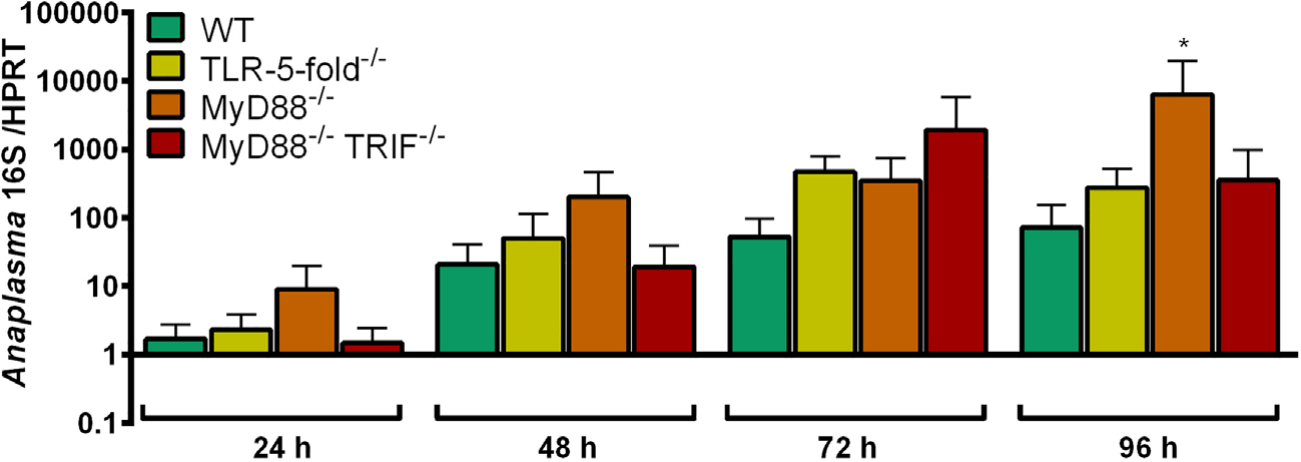
Increase of *A. phagocytophilum* 16S rRNA relative to murine HPRT mRNA at different time points p.i. in WT, TLR-5-fold^-/-^, MyD88^-/-^, and MyD88^-/-^ TRIF^-/-^ Hoxb8 neutrophils. Results were normalized to the respective 0 h value of each sample using the ΔΔC_t_-method. Mean and SD from five independent experiments are shown. Differences between experimental groups were analyzed using the two-tailed Mann–Whitney test. The following groups were compared: WT and the respective gene-deficient Hoxb8 neutrophils at each time point. *p < 0.05.

The growth of *A. phagocytophilum* in MyD88^-/-^, MyD88^-/-^ TRIF^-/-^ and TLR-5-fold^-/-^ Hoxb8 neutrophils was compared to WT cells. We hypothesized that *A. phagocytophilum* would replicate better in gene-deficient cells if it stimulates neutrophils *via* the respective PRR. However, except for MyD88^-/-^ Hoxb8 neutrophils at 96 h p.i. (*p* < 0.05), no significant differences in bacterial growth were found (**Figure 2**).

#### iNOS mRNA Induction in TLR-5-fold^-/-^, MyD88^-/-^, and MyD88^-/-^ TRIF^-/-^ Hoxb8 Neutrophils

The infection of Hoxb8 neutrophils with *A. phagocytophilum* has been shown previously to induce iNOS mRNA (Gussmann et al., 2017). Therefore, this parameter was analyzed here. LPS was used as control stimulus.

**Figure 3:**
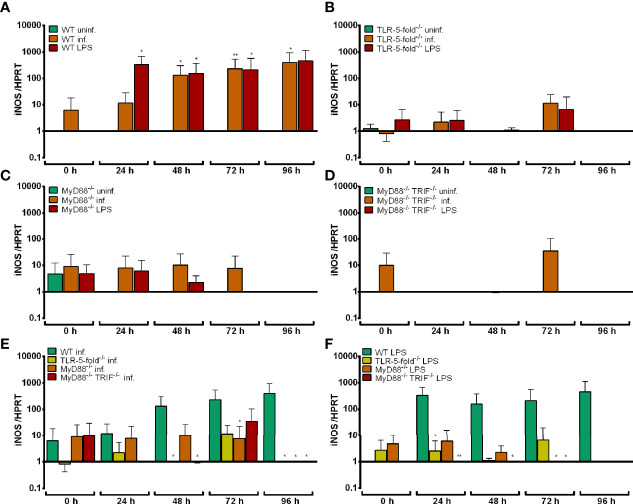
Relative iNOS mRNA expression normalized to murine HPRT at different time points in uninfected, *A*. *phagocytophilum* infected and LPS-stimulated WT **(A)**, TLR-5-fold^-/-^ **(B)**, MyD88^-/-^ **(C)** and MyD88^-/-^ TRIF^-/-^ **(D)** Hoxb8 neutrophils. Infected **(E)** and LPS-stimulated **(F)** WT and gene-deficient Hoxb8 neutrophils were compared side by side in panels **(E)** and **(F)**. Results were normalized to the 0 h value of uninfected WT cells using the ΔΔC_t_-method. Mean and SD from five independent experiments are shown. Differences between experimental groups were analyzed using the two-tailed Mann–Whitney test. The following groups were compared: WT, TLR-5-fold^-/-^, MyD88^-/-^, and MyD88^-/-^ TRIF^-/- ^*A. phagocytophilum* infected or LPS-stimulated Hoxb8 neutrophils to the respective uninfected control cells at each time point **(A–D)**, *A. phagocytophilum* infected or LPS-stimulated TLR-5-fold^-/-^, MyD88^-/-^, and MyD88^-/-^ TRIF^-/-^ Hoxb8 neutrophils to *A. phagocytophilum* infected or LPS-stimulated WT cells **(E, F)**. *p < 0.05, **p < 0.01.

As shown in **Figure 3A**, iNOS mRNA expression was significantly induced in WT Hoxb8 neutrophils after *A. phagocytophilum* infection or LPS-stimulation at 24 h to 96 h p.i. compared to uninfected control cells (*p* < 0.05). In contrast, iNOS mRNA was not up-regulated upon infection or LPS-stimulation in TLR-5-fold^-/-^ (**Figure 3B**), MyD88^-/-^ (**Figure 3C**) and MyD88^-/-^ TRIF^-/-^ (**Figure 3D**) Hoxb8 neutrophils compared to the respective uninfected or unstimulated control cells. However, the differences in iNOS mRNA expression between WT and gene-deficient *A. phagocytophilum* infected cells reached statistical significance at most, but not all time points (**Figure 3E**). The same was true for LPS-stimulation (**Figure 3F**).

#### Chemokine and Cytokine Secretion of TLR-5-fold^-/-^, MyD88^-/-^, and MyD88^-/-^ TRIF^-/-^ Hoxb8 Neutrophils

The infection of Hoxb8 neutrophils with *A. phagocytophilum* has been shown previously to induce the secretion of MIP-1α, RANTES and TNF (Gussmann et al., 2017). Therefore, these parameters were analyzed here. LPS was used as control stimulus.

Statistically significant higher amounts of MIP-1α (*p* < 0.01) (**Figure S1A**), RANTES (*p* < 0.05) (**Figure S1B**) and TNF (24–48 h p.i. *p* < 0.01, 72–96 h p.i. *p* < 0.05) (**Figure S1C**) were found in the supernatants of *A. phagocytophilum*-infected or LPS-stimulated WT Hoxb8 neutrophils compared to the medium controls.

Significantly lower amounts of MIP-1-α (**Figure S2A**), RANTES (**Figure S2B**) and TNF (**Figure S2C**) were detected in the supernatants of TLR-5-fold^-/-^, MyD88^-/-^ and MyD88^-/-^ TRIF^-/-^ Hoxb8 neutrophils at 24–96 h p.i. compared to WT control cells after LPS-stimulation (*p* < 0.01). This is consistent with the absence of LPS-signaling *via* TLR4, MyD88 and TRIF in the respective gene-deficient Hoxb8 neutrophils (Takeda and Akira, 2004).

**Figure 4:**
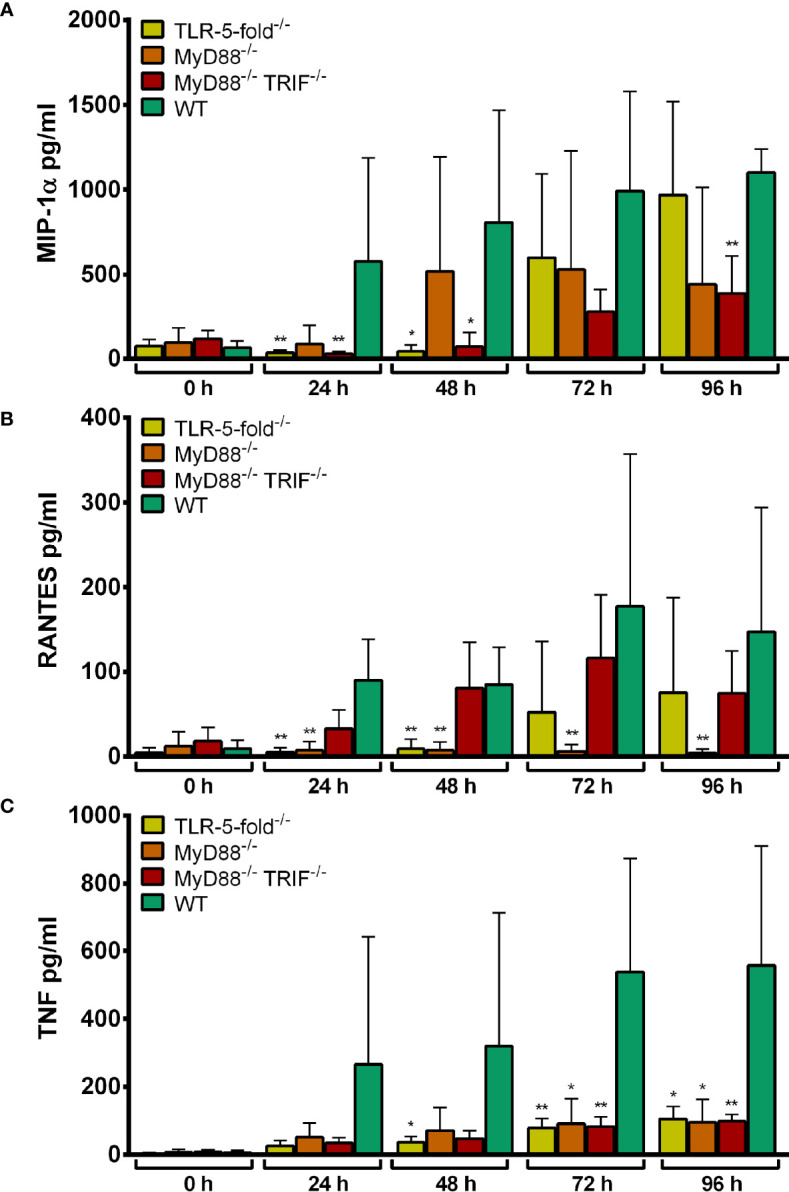
MIP-1α **(A)**, RANTES **(B)** and TNF **(C)** production in TLR-5-fold^-/-^, MyD88^-/-^, MyD88^-/-^ TRIF^-/-^ and WT Hoxb8 neutrophils at different time points after infection with *A. phagocytophilum*. MIP-1α, RANTES and TNF were measured in the supernatants using CBA assay. Mean and SD from five **(A, C)** and four **(B)** independent experiments are shown, respectively. Differences between the respective gene-deficient Hoxb8 neutrophils and WT control cells at each time point were analyzed using the two-tailed Mann–Whitney test. *p < 0.05, **p < 0.01.

Upon infection with *A. phagocytophilum*, a similar finding was made for TNF (**Figure 4C**). For MIP-1α (**Figure 4A**) and RANTES (**Figure 4B**) production, the differences between WT and gene-deficient Hoxb8 neutrophils were less uniform at the different time points after infection.

#### Conclusion

Together, these data suggest that *in vitro A. phagocytophilum* might be recognized by Hoxb8 neutrophils *via* MyD88-dependent TLR-signaling.

### TLR7-, TLR9-, and TRIF-Dependent Recognition of *A. phagocytophilum*

The TLR-5-fold^-/-^ Hoxb8 neutrophils used here were deficient for TLR2, 3, 4, 7, and 9. A*. phagocytophilum* lacks the established ligands for TLR3 (dsRNA) and TLR4 (LPS) (Lin and Rikihisa, 2003; Otten et al., 2018). Therefore, TLR7- and TLR9-dependent signaling were estimated as the most likely options and investigated using single gene-deficient cells. We also included TRIF^-/-^ Hoxb8 neutrophils because *in vivo* TRIF-deficient mice had a significantly elevated bacterial load in blood at day 3 p.i (**Figure 1A**).

#### Growth of *A. phagocytophilum* in TLR-7^-/-^, TLR9^-/-^, and TRIF^-/-^ Hoxb8 Neutrophils

**Figure 5:**
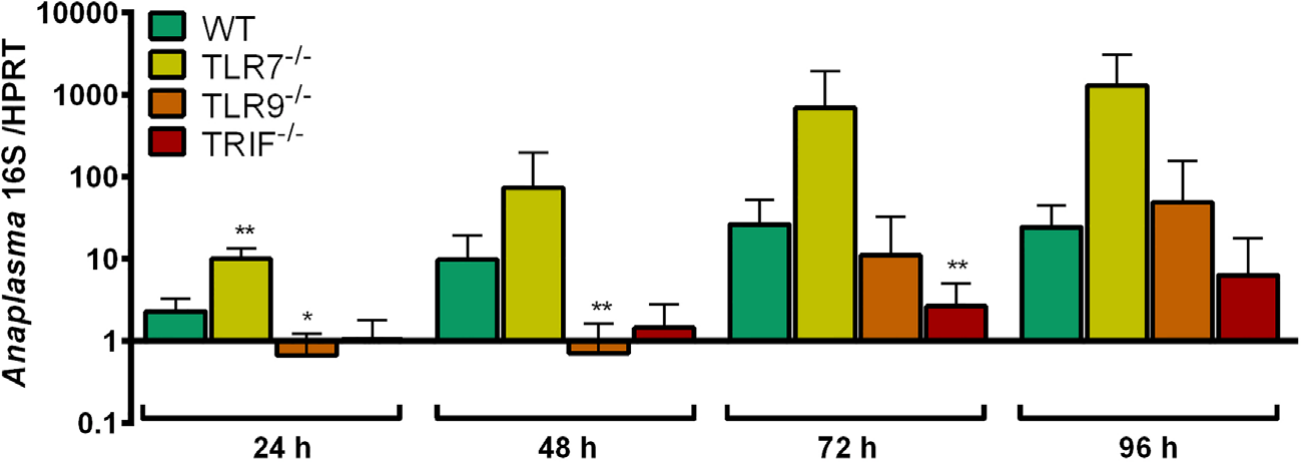
Increase of *A. phagocytophilum* 16S rRNA relative to murine HPRT mRNA at different time points p.i. in WT, TLR7^-/-^, TLR9^-/-^ and TRIF^-/-^ Hoxb8 neutrophils. Results were normalized to the respective 0 h value of each sample using the ΔΔC_t_-method. Mean and SD from five independent experiments are shown. Differences between experimental groups were analyzed using the two-tailed Mann–Whitney test. The following groups were compared: WT and the respective gene-deficient Hoxb8 neutrophils at each time point. *p < 0.05, **p < 0.01.

The growth of *A. phagocytophilum* in TLR7^-/-^, TLR9^-/-^ and TRIF^-/-^ Hoxb8 neutrophils was compared to WT cells. At all time-points analyzed, *A. phagocytophilum* grew better in TLR7^-/-^ Hoxb8 neutrophils than in WT cells. However, the difference was only significant at 24 h p.i. (**Figure 5**). In TLR9^-/-^ and TRIF^-/-^ Hoxb8 neutrophils, the replication of *A. phagocytophilum* was worse or equal to WT cells.

#### iNOS mRNA Induction in TLR-7^-/-^, TLR9^-/-^, and TRIF^-/-^ Hoxb8 Neutrophils

The TLR7 agonist imiquimod was used as control stimulus for TLR7-dependent signaling, the CpG oligonucleotide ODN 1826 for TLR9-dependent signaling and LPS for TRIF-dependent signaling.

Imiquimod induced iNOS mRNA expression in WT Hoxb8 cells (**Figure S3A**). The iNOS mRNA expression in TLR7^-/-^ Hoxb8 neutrophils after imiquimod-stimulation was significantly lower than in WT cells at 24 and 48 h p.i. (*p* < 0.05) proving the specificity of the effect (**Figure S3B**).

In contrast, ODN 1826 did not significantly induce iNOS mRNA expression in WT Hoxb8 cells (**Figure S3A**). In line with that finding, there was no statistically significant difference between iNOS mRNA expression in WT and TLR9^-/-^ Hoxb8 neutrophils after stimulation with ODN 1826 (**Figure S3C**). This indicates that TLR9 signaling alone is insufficient for iNOS induction in Hoxb8 WT neutrophils.

LPS induced iNOS mRNA expression in WT Hoxb8 cells (**Figure S3A**). However, iNOS mRNA expression in TRIF^-/-^ Hoxb8 neutrophils was unaltered compared to WT cells after LPS-stimulation (**Figure S3D**). This is explained best by the MyD88-dependent TLR4 signaling which is still active in TRIF-deficient myeloid cells (Takeda and Akira, 2004).

**Figure 6:**
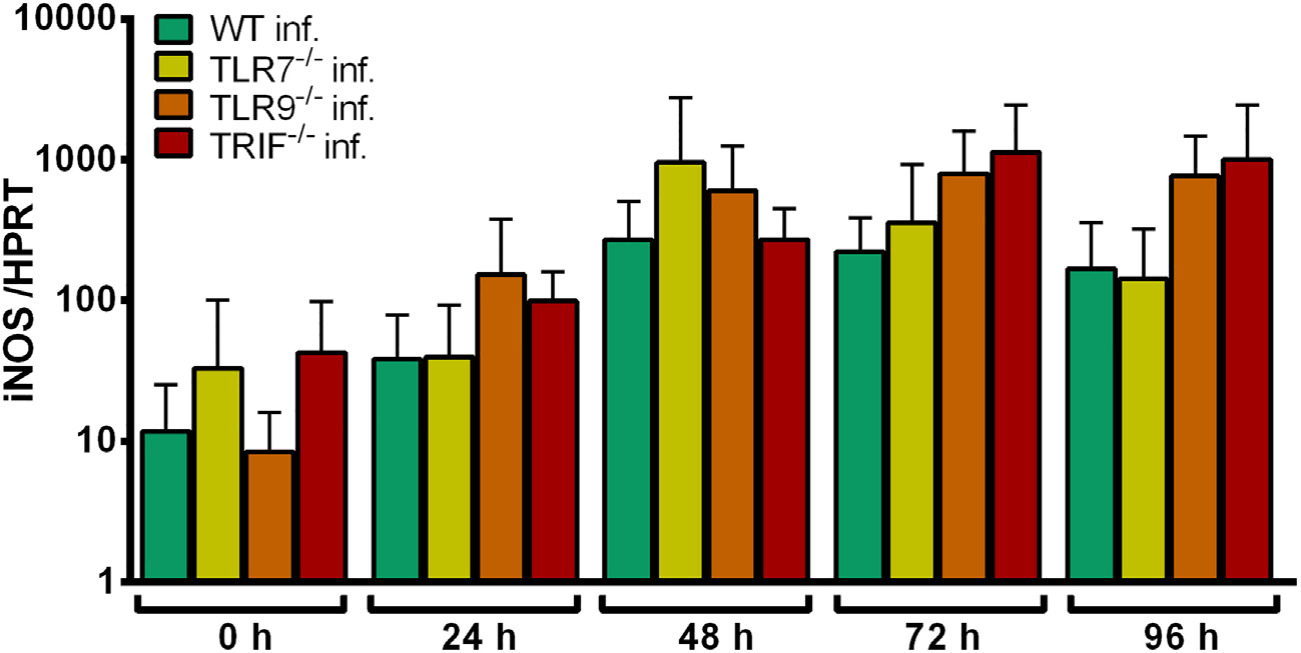
Relative iNOS mRNA expression normalized to murine HPRT in WT, TLR7^-/-^, TLR9^-/-^ and TRIF^-/-^ Hoxb8 neutrophils at different time points after infection with *A. phagocytophilum*. Results were normalized to the 0 h value of uninfected WT cells using the ΔΔC_t_-method. Mean and SD from five independent experiments are shown. Differences between experimental groups were analyzed using the two-tailed Mann–Whitney test. The following groups were compared: the respective *A. phagocytophilum* infected gene-deficient Hoxb8 neutrophils to WT control cells.

iNOS induction in WT, TLR7^-/-^, TLR9^-/-^, and TRIF^-/-^ Hoxb8 neutrophils after infection with *A. phagocytophilum* was not significantly different (**Figure 6**). This argues against TLR7-dependent recognition of *A. phagocytophilum*. iNOS mRNA expression could not be used as a readout for TLR9^-/–^dependent signaling because it was not induced after ODN 1826-stimulation (**Figures S3A, C**)

#### Chemokine and Cytokine Secretion of TLR-7^-/-^, TLR9^-/-^, and TRIF^-/-^ Hoxb8 Neutrophils

In addition to MIP-1-α, RANTES and TNF, IL-6 was included in the analysis of chemokine and cytokine production because human neutrophils produced IL-6 after ODN 1826-stimulation (József et al., 2006).

Imiquimod significantly induced the production of MIP-1α at 24–48 h (*p* < 0.01) and at 96 h (*p* < 0.05) p.i. in WT Hoxb8 neutrophils compared to unstimulated control cells (**Figure S4A**), but failed to stimulate the secretion of relevant amounts of RANTES (**Figure S4B**), TNF (**Figure S4C**) and IL-6 (**Figure S4D**).

In contrast, ODN 1826- or LPS-stimulation led to significantly elevated levels of MIP-1α (**Figure S4A**), RANTES (**Figure S4B**), TNF (**Figure S4C**) and IL-6 (**Figure S4D**) in WT Hoxb8 neutrophils at 24–96 h p.i. compared to unstimulated control cells (*p* < 0.01).

TLR7^-/-^ Hoxb8 neutrophils produced significantly lower amounts of MIP-1α (**Figure S5A**), RANTES (**Figure S5B**) and TNF (**Figure S5C**) than WT control cells after stimulation with imiquimod. However in contrast to MIP-1α (µ = 229 pg/ml at 24 h), the amounts of RANTES (µ = 11 pg/ml at 24 h) and TNF (µ = 33 pg/ml at 24 h) in the supernatants of WT Hoxb8 neutrophils after stimulation with imiquimod were very low. IL-6 secretion of WT and TLR7^-/-^ Hoxb8 neutrophils was not significantly different at all but one time points. (**Figure S5D**).

TLR9^-/-^ Hoxb8 neutrophils produced significantly less MIP-1α (**Figure S6A**), TNF (**Figure S6C**) and IL-6 (**Figure S6D**) at 24–96 h p.i. after stimulation with ODN 1826 than WT control cells (*p* < 0.01). In the case of RANTES, a significant difference was only observed at 24 h p.i. (*p* < 0.01) (**Figure S6B**).

The supernatants of TRIF^-/-^ Hoxb8 neutrophils contained significantly lower amounts of RANTES than WT control cells at 24–96 h p.i. after LPS-stimulation (*p* < 0.01) (**Figure S7B**). This was not found for MIP-1α (**Figure S7A**), TNF (**Figure S7C**) and IL-6 (**Figure S7D**),where TRIF^-/-^ and WT Hoxb8 neutrophils showed comparable values at most time points.

According to these data, MIP-1α production seems to be a suitable readout for TLR7-dependent signaling in Hoxb8 neutrophils. Correspondingly, MIP-1α, TNF and IL-6 were found appropriate to investigate immune stimulation *via* TLR9. For the TRIF-pathway RANTES was selected.

**Figure 7:**
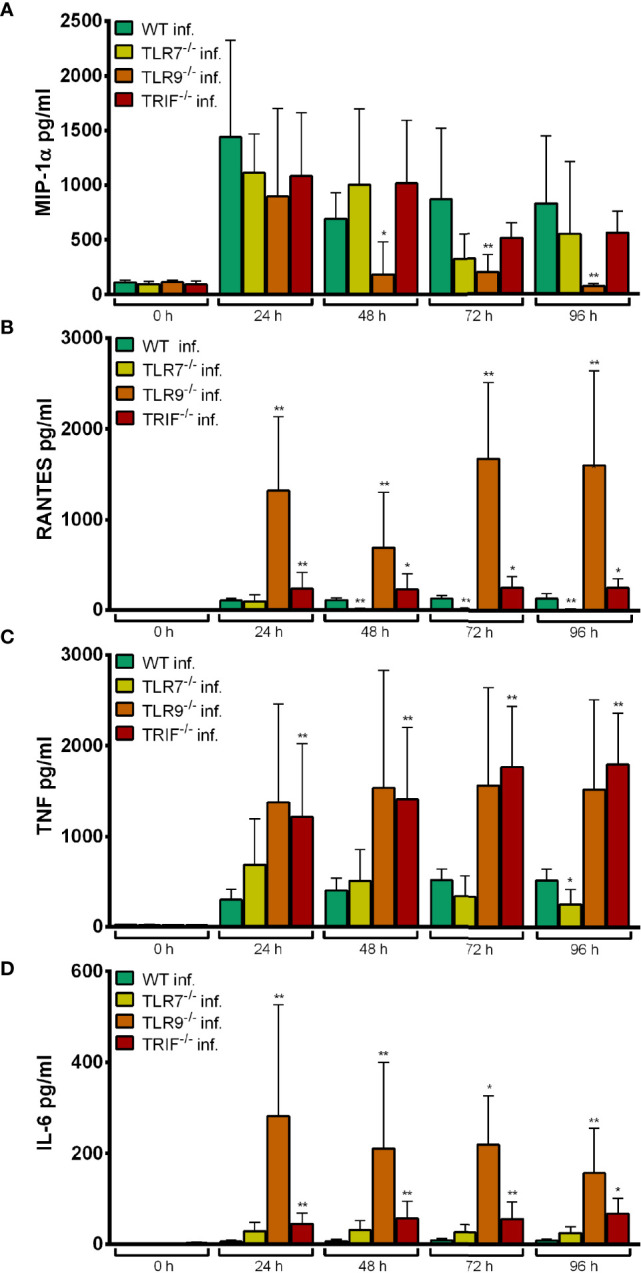
MIP-1α **(A)**, RANTES **(B)**, TNF **(C)** and IL-6 **(D)** production in WT, TLR7^-/-^, TLR9^-/-^ and TRIF^-/-^ Hoxb8 neutrophils at different time points after *A. phagocytophilum* infection. MIP-1α, RANTES, TNF and IL-6 were measured in the supernatants using CBA assay. Mean and SD from five independent experiments are shown. Differences between the respective gene-deficient Hoxb8 neutrophils and WT control cells at each time point were analyzed using the two-tailed Mann–Whitney test. *p < 0.05, **p < 0.01.

MIP-1α production upon infection with *A. phagocytophilum* was unaltered in TLR7^-/-^ Hoxb8 neutrophils compared to WT cells (**Figure 7A**).

In contrast, TLR9^-/-^ Hoxb8 neutrophils secreted significantly less MIP-1α at 48 h (*p* < 0.05) and 72–96 h (*p* < 0.01) p.i. than WT cells (**Figure 7A**). The TNF production of TLR9^-/-^ Hoxb8 neutrophils after *A. phagocytophilum* infection was not different from WT cells (**Figure 7C**). However, RANTES (**Figure 7B**) and IL-6 (**Figure 7D**) secretion of TLR9^-/-^ Hoxb8 neutrophils was significantly increased compared to WT cells after 24 h – 96 h p.i. (*p* < 0.01).

TRIF^-/-^ Hoxb8 neutrophils secreted significantly more RANTES (**Figure 7B**), TNF (**Figure 7C**) and IL-6 (**Figure 7D**) than WT cells upon infection.

The elevated chemokine and cytokine production in TLR9^-/-^ (RANTES, IL-6) and TRIF^-/-^ (RANTES, TNF, IL-6) Hoxb8 neutrophils (**Figure 7**) might represent a compensation mechanism.

#### Conclusion

We had identified lower iNOS mRNA expression and reduced MIP-1α production as readout for TLR7-dependent signaling in Hoxb8 neutrophils, but did not see an effect upon *A. phagocytophilum* infection (**Figures 6**, **7A**). Therefore *in vitro*, we did not find evidence for immune recognition of *A. phagocytophilum via* TLR7.

MIP-1α, TNF, and IL-6 were the three parameters chosen for investigation of TLR9 activation. Significantly lower amounts in the supernatants of the gene-deficient Hoxb8 neutrophils were only found for MIP1-α and only at later time points of 48–96 h p.i. (**Figure 7A**) Thus, TLR9-dependent recognition of *A. phagocytophilum* could not be convincingly proven.

*In vivo*, TLR7^-/-^ and TLR9^-/-^ mice were able to control *A. phagocytophilum* as efficiently as WT mice (**Figure S8**).

### NLR-Dependent Recognition of *A. phagocytophilum* in Neutrophils *In Vitro*

We decided to study the NLR pathway using as representatives NOD1, NOD2, and NALP3 (NLRP3), because *in vivo* mice with defective signaling *via* TLR were still able to control *A. phagocytophilum* as efficiently as WT animals (von Loewenich et al., 2004; Pedra et al., 2007b).

#### Growth of *A. phagocytophilum* in NOD1^-/-^, NOD2^-/-^, and NALP3^-/-^ Hoxb8 Neutrophils

**Figure 8:**
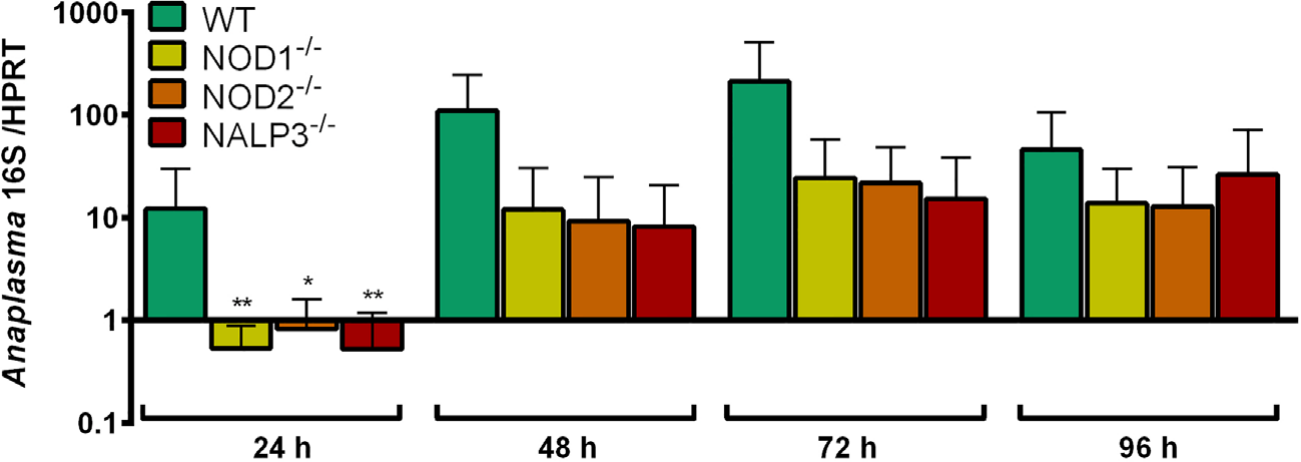
Increase of *A. phagocytophilum* 16S rRNA relative to murine HPRT mRNA at different time points p.i. in WT, NOD1^-/-^, NOD2^-/-^, and NALP3^-/-^ Hoxb8 neutrophils. Results were normalized to the respective 0 h value of each sample using the ΔΔC_t_-method. Mean and SD from four independent experiments are shown. Differences between experimental groups were analyzed using the two-tailed Mann–Whitney test. The following groups were compared: WT and the respective gene-deficient Hoxb8 neutrophils at each time point. *p < 0.05 **p < 0.01.

The growth of *A. phagocytophilum* in NOD1^-/-^, NOD2^-/-^, and NALP3^-/-^ Hoxb8 neutrophils was not significantly better than in WT control cells (**Figure 8**).

#### iNOS mRNA Induction in NOD1^-/-^, NOD2^-/-^, and NALP3^-/-^ Hoxb8 Neutrophils

γ-D-glutamyl-meso-diaminopimelic acid (iE-DAP) was used as control agonist for NOD1 and muramyldipeptide (MDP) as control agonist for NOD2 and NALP3. iE-DAP and MPD did not induce iNOS mRNA expression in WT Hoxb8 neutrophils (data not shown). Upon infection with *A. phagocytophilum*, statistically significant differences in iNOS mRNA expression between NOD1^-/-^, NOD2^-/-^, NALP3^-/-^ and WT Hoxb8 neutrophils were not found (data not shown).

#### Chemokine and Cytokine Secretion of NOD1^-/-^, NOD2^-/-^, and NALP3^-/-^ Hoxb8 Neutrophils

MIP-1α, RANTES, KC, TNF, IL-6, and IL-1β were measured in the supernatants of gene-deficient and WT control Hoxb8 neutrophils upon stimulation with iE-DAP or MDP. However, iE-DAP and MDP did not induce the expression of any of the chemokines or cytokines tested (data not shown).

**Figure 9:**
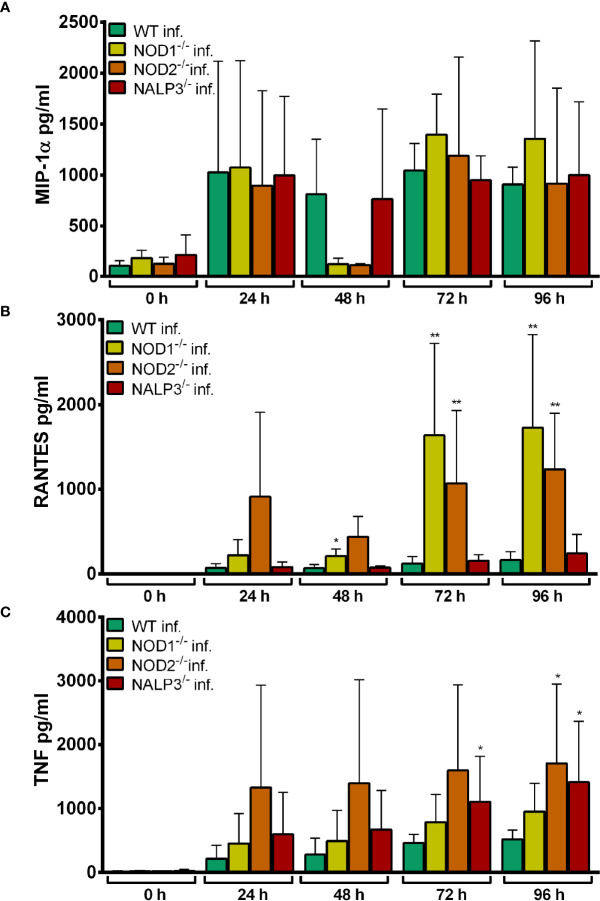
MIP-1α **(A)**, RANTES **(B)** and TNF **(C)** production in WT, NOD1^-/-^, NOD2^-/-^, and NALP3^-/-^ Hoxb8 neutrophils at different time points after *A*. *phagocytophilum* infection. MIP-1α, RANTES and TNF were measured in the supernatants using CBA assay. Mean and SD from five independent experiments are shown. Differences between the respective gene-deficient Hoxb8 neutrophils and WT control cells at each time point were analyzed using the two-tailed Mann–Whitney test. *p < 0.05, **p < 0.01.

The production of MIP-1α (**Figure 9A**), RANTES (**Figure 9B**) and TNF (**Figure 9C**) was not significantly lower in the gene-deficient Hoxb8 neutrophils than in WT control cells upon *A. phagocytophilum* infection.

#### Conclusion

Together these data suggest that at least in Hoxb8 neutrophils signaling *via* NOD1, NOD2, and NALP3 seems not to be involved in immune recognition of *A. phagocytophilum*.

### CLR-Dependent Recognition of *A. phagocytophilum* in Neutrophils *In Vitro*

Next, we investigated whether the adaptor/signaling proteins of different CLR such as DAP12, FcRγ and SYK (Brown et al., 2018) were involved in the stimulation of Hoxb8 neutrophils by *A. phagocytophilum*. SYK-deficient polyclonal progenitor cell lines were severely impaired in growth. Therefore, SYK^del/del-^ Hoxb8 neutrophils could not be produced in significant amounts and were not investigated further.

#### Growth of *A. phagocytophilum* in DAP12^-/-^ and FcRγ^-/-^ Hoxb8 Neutrophils

**Figure 10:**
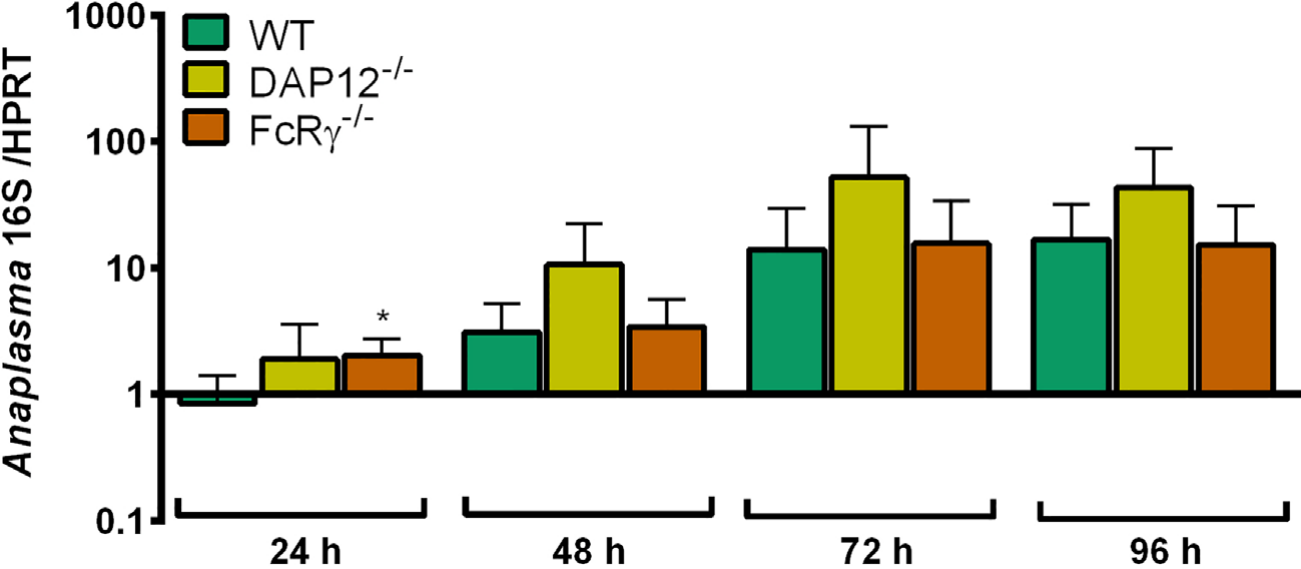
Increase of *A. phagocytophilum* 16S rRNA relative to murine HPRT mRNA at different time points p.i. in WT, DAP12^-/-^ and FcRγ^-/-^ Hoxb8 neutrophils. Results were normalized to the respective 0 h value of each sample using the ΔΔC_t_-method. Mean and SD from five independent experiments are shown. Differences between experimental groups were analyzed using the two-tailed Mann–Whitney test. The following groups were compared: WT and the respective gene-deficient Hoxb8 neutrophils at each time point. *p < 0.05.

*A. phagocytophilum* did not replicate significantly better in DAP12^-/-^ and FcRγ^-/-^ Hoxb8 neutrophils than in WT cells except for 24 h p.i. in FcRγ^-/-^ Hoxb8 neutrophils (*p* < 0.05) (**Figure 10**).

#### iNOS mRNA Induction in DAP12^-/-^ and FcRγ^-/-^ Hoxb8 Neutrophils

It is not known for all CLR, which molecular pattern they bind. However, mycobacteria are recognized by some of them (Geijtenbeek and Gringhuis, 2009; Brown et al., 2018). Therefore, we used heat-killed mycobacteria (HKMT) as control stimulus and compared it to LPS.

HKMT, LPS and *A. phagocytophilum* significantly induced iNOS mRNA expression in WT (**Figure S9A**), DAP12^-/-^ (**Figure S9B**) and FcRγ^-/-^ (**Figure S9C**) Hoxb8 neutrophils at 24–72 h p.i. iNOS induction might not be necessarily diminished in DAP12- or FcRγ-deficient cells after HKMT-stimulation because HKMT possess other PRR ligands for example for TLR2 (Underhill et al., 1999).

**Figure 11:**
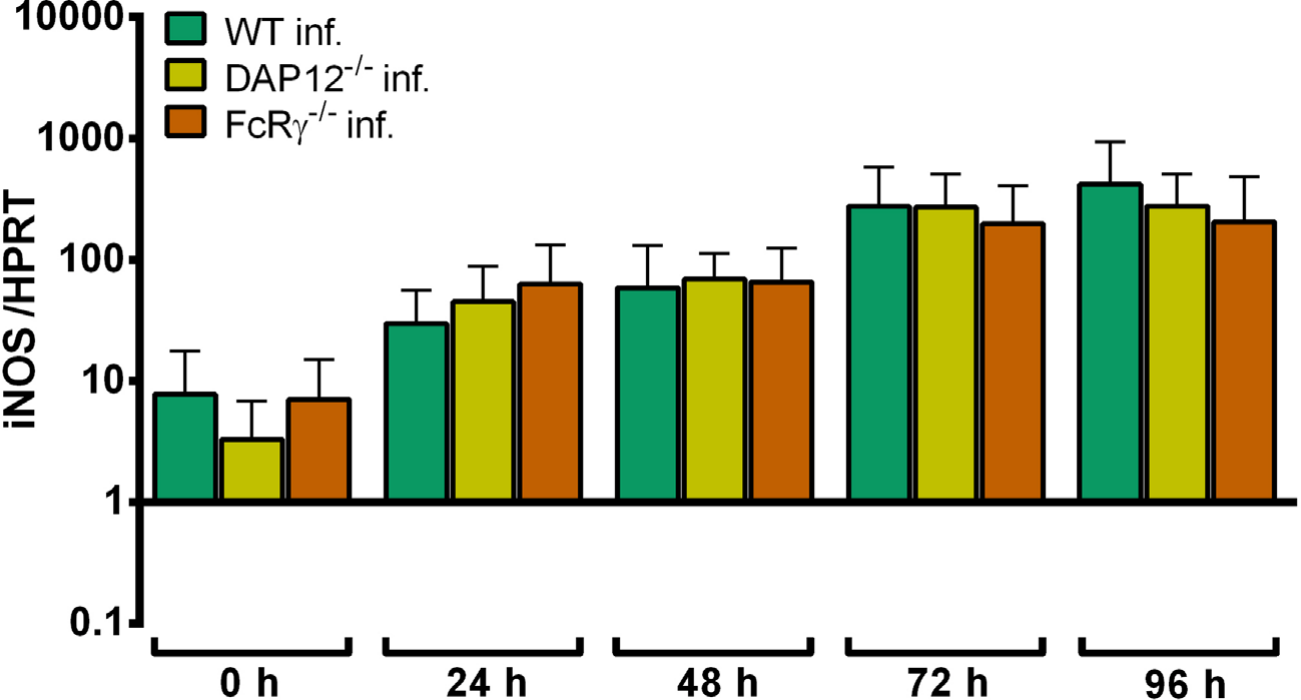
Relative iNOS mRNA expression normalized to murine HPRT at different time points in *A. phagocytophilum* infected WT, DAP12^-/-^and FcRγ^-/-^ Hoxb8 neutrophils. Results were normalized to the 0 h value of uninfected WT cells using the ΔΔC_t_-method. Mean and SD from 5 independent experiments are shown. Differences between gene-deficient Hoxb8 neutrophils and WT control cells at each time point were analyzed using the two-tailed Mann–Whitney test.

Upon infection with *A. phagocytophilum*, iNOS mRNA was expressed similarly in DAP12^-/-^, FcRγ^-/-^ and WT Hoxb8 neutrophils (**Figure 11**).

#### Chemokine and Cytokine Secretion of DAP12^-/-^ and FcRγ^-/-^ Hoxb8 Neutrophils

Significant higher amounts of MIP-1α (**Figure S10A**), RANTES (**Figure S10B**), and TNF (**Figure S10C**) were produced by WT Hoxb8 neutrophils after HKMT- and LPS-stimulation or *A. phagocytophilum* infection compared to medium controls at 24–96 h p.i. For IL-6, this was only found for treatment with HKMT or LPS, but not for infection with *A. phagocytophilum* (**Figure S10D**).

In essence, the amount of MIP-1α, RANTES, TNF and IL-6 in the supernatants of DAP12^-/-^ and FcRγ^-/-^ Hoxb8 neutrophils was not significantly different to WT control cells after stimulation with HKMT (**Figure S11**) or LPS (**Figure S12**). However, DAP12^-/-^ Hoxb8 neutrophils produced significantly more RANTES (**Figures S11B**, **S12B**) and IL-6 (**Figures S11D**, **12D**) than WT cells after HKMT- or LPS-treatment at 24–96 h. A similar observation was made earlier for murine DAP12^-/-^ bone marrow derived DC (Chu et al., 2008) and murine DAP12^-/-^ murine bone marrow derived macrophages (Hamerman et al., 2005) that produced significantly higher amounts of IL-6 than WT control cells after LPS-stimulation which was interpreted as negative regulation of the TLR-response by DAP12. Here, we show that this is also true for Hoxb8 neutrophils.

**Figure 12:**
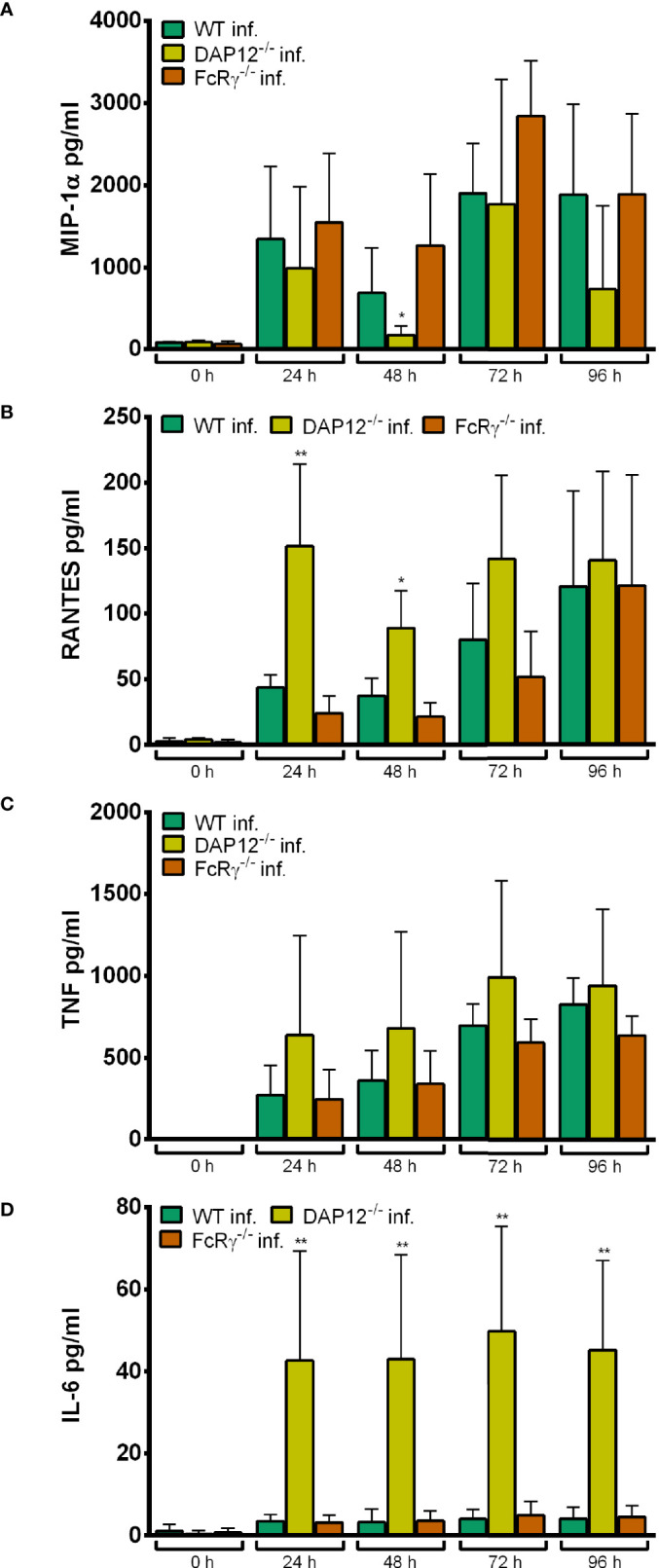
MIP-1α **(A)**, RANTES **(B)**, TNF **(C)** and IL-6 **(D)** production in WT, DAP12^-/-^ and FcRγ^-/-^ Hoxb8 neutrophils at different time points after infection with *A*. *phagocytophilum*. MIP-1α, RANTES, TNF and IL-6 were measured in the supernatants using CBA assay. Mean and SD from five independent experiments are shown. Differences between the respective gene-deficient Hoxb8 neutrophils and WT control cells at each time point were analyzed using the two-tailed Mann–Whitney test. *p < 0.05, **p < 0.01.

In essence, DAP12^-/-^ and FcRγ^-/-^ Hoxb8 neutrophils produced similar amounts of MIP-1α (**Figure 12A**), RANTES (**Figure 12B**) and TNF (**Figure 12C**) upon infection with *A. phagocytophilum*. In contrast, significantly higher amounts of IL-6 were found in the supernatants of infected DAP12^-/-^ Hoxb8 neutrophils than in WT cells at 24–96 h p. i. (*p* < 0.01) (**Figure 12D**).

#### Conclusion

Together these data suggest that CLR signaling *via* DAP12 or FcRγ seems not to be involved in immune recognition of *A. phagocytophilum*, because it did not grow significantly better in the gene-deficient Hoxb8 neutrophils and because the chemokine and cytokine response was not significantly lower than in WT cells.

## Discussion

### TLR-Dependent Recognition of *A. phagocytophilum*

Neutrophil granulocytes express a wide variety of PRR (Thomas and Schroder, 2013). In murine neutrophils, the expression of TLR1, TLR2, TLR4, TLR5, TLR7, and TLR9 has been reported (Thomas and Schroder, 2013). It has been shown primarily in macrophages that the activation of TLR2, TLR3, TLR4, TLR5, and TLR9 mediates iNOS induction and NO production (Abdul-Cader et al., 2016). We found here that iNOS mRNA in murine Hoxb8 neutrophils was induced in a TLR- and MyD88-dependent manner after LPS-stimulation and in a TLR7-dependent manner after imiquimod-stimulation. In contrast, iNOS expression was unaltered when Hox8 neutrophils were exposed to TLR9 agonist ODN 1826. Previously, it has been shown that the infection of murine bone marrow derived neutrophils with *Leishmania infantum* induced iNOS mRNA expression and NO production in a TLR2-dependent manner (Sacramento et al., 2017). Further, zymosan, a cell wall component of yeasts, triggered iNOS protein synthesis and NO release of murine peripheral blood neutrophils *via* TLR2 and MyD88 (Li et al., 2016). To our knowledge, TLR7-dependent iNOS induction in neutrophils has not been shown before. However in murine monocytes, iNOS expression was partially TLR7-dependent after chikungunya virus infection (McCarthy et al., 2020).

In murine neutrophils, the expression of MIP-1α, RANTES, TNF, and IL-6 has been reported (Tecchio et al., 2014). We showed here that MIP-1α, RANTES and TNF were secreted by murine Hoxb8 neutrophils in a TLR- and MyD88-dependent manner after LPS-stimulation. MIP-1α production was induced *via* TLR7 after incubation with imiquimod and MIP-1α, TNF and IL-6 production *via* TLR9 after incubation with ODN 1826. Previously, it has been shown that secretion of TNF (Mohammadi et al., 2016), KC (CXCL1) and MIP-2 (CXCL2) (Lentini et al., 2020) by murine bone marrow derived neutrophils after stimulation with *Streptococcus agalactiae* was diminished in the absence of the endosomal TLR7, 9 and 13, whereas the lack of the individual TLR had no effect.

Here, we provide evidence that *A. phagocytophilum* is recognized *via* TLR- and MyD88-dependent signaling by neutrophils, because iNOS mRNA induction and TNF production were significantly lower in Hoxb8 neutrophils that lacked simultaneously TLR2, 3, 4, 7 and 9 as well as in Hoxb8 neutrophils singly defective for MyD88. *A. phagocytophilum* does not possess the established ligands for TLR3 (dsRNA) and TLR4 (LPS) (Lin and Rikihisa, 2003; Otten et al., 2018). Therefore, TLR7- and TLR9-dependent signaling were estimated as the most likely options and investigated using single gene-deficient cells. However, convincing evidence for recognition of *A. phagocytophilum via* TLR7 and TLR9 by Hoxb8 neutrophils was not provided. A reason for this could be that *A. phagocytophilum* expresses ligands for TLR7 and TLR9 and that the effect might be seen only in double deficient cells. In murine peritoneal macrophages, activation of NK-κB after stimulation with *A. phagocytophilum* was triggered *via* TLR2, but was independent of TLR4 (Choi et al., 2004 ). Thus, the effect we saw in Hoxb8 neutrophils defective for TLR2, 3, 4, 7 and 9 could be mediated by TLR2.

*Ehrlichia chaffeensis* and *E. muris* are obligate intracellular bacteria related to *A. phagocytophilum* that replicate in monocytes and macrophages (Dumler et al., 2001). After infection with *E. chaffeensis*, MIP-2 (CXCL2) and TNF mRNA induction in murine bone marrow derived macrophages was MyD88-dependent, but did not require TLR2 or TLR4 (Miura et al., 2011). Similarly, IL-12 production of bone marrow derived DC after stimulation with *E. muris* was reduced in the absence of MyD88 (Koh et al., 2010). However, the effect was independent of TLR2, 3, 4, 5, 7, 9, 11, IL-1 and IL-18 signaling. Maybe, as discussed above, the lack of one single TLR is compensated by the others. In contrast to these findings, IFN-γ expression of murine spleen cells after incubation with *E. muris* required the antigen-presenting molecule CD1d, but was MyD88-independent (Mattner et al., 2005).

*In vitro*, the growth of *A. phagocytophilum* in Hoxb8 neutrophils defective for TLR2, 3, 4, 7, 9 and MyD88 was not essentially altered. This suggests that the pathogen escapes the immune reaction mediated by neutrophils without additional stimulation. Protection of *A. phagocytophilum* against reactive oxygen species has been shown before (Carlyon et al., 2004; IJdo and Mueller, 2004). However when Hoxb8 neutrophils were stimulated by IFN-γ, the growth of *A. phagocytophilum* was significantly impaired (Gussmann et al., 2017). *In vivo*, mice deficient for TLR2, TLR4 and MyD88 were fully able to control *A. phagocytophilum* (von Loewenich et al., 2004; Pedra et al., 2007b). In contrast, mice deficient for the co-stimulatory molecule CD40 experienced persistent infection and the depletion of CD11c^+^ DC delayed pathogen clearance (Birkner et al., 2008). Therefore, in antigen-presenting cells MyD88-independent PRR pathways might be involved in the recognition of *A. phagocytophilum* or *in vivo* the defect is compensated because of the redundancy of the immune system. In contrast to *A. phagocytophilum*, *E. muris* infected MyD88^-/-^ mice had elevated bacterial loads in blood and spleen at days 10 and 14 p.i. compared to WT animals probably because of an impaired IFN-γ response of CD4^+^ T cells following reduced IL-12 production by DC (Koh et al., 2010).

### NLR-Dependent Recognition of *A. phagocytophilum*

NOD1 (Jeong et al., 2014), NOD2 (Jeong et al., 2014) and NALP3 (Guarda et al., 2011) protein expression has been shown in murine neutrophils. To our knowledge, no data exist whether NLR stimulation induces iNOS in neutrophils. Here, iNOS mRNA was not upregulated in Hoxb8 neutrophils after stimulation with the NOD agonists iE-DAP or MDP. However, MDP-stimulation of human peripheral blood mononuclear cells led to iNOS mRNA and protein expression in a NOD2-dependent manner (Landes et al., 2015).

Here, MIP-1α, RANTES, KC, TNF, IL-6 and IL-1β were not secreted by murine Hoxb8 neutrophils after incubation with iE-DAP or MDP. Previously, it has been shown that murine thioglycollate-elicited peritoneal exudate neutrophils produced MCP-1 (CCL2), KC (CXCL1), TNF and IL-6 after MDP-stimulation in a NOD2-dependent manner (Jeong et al., 2014). This was not the case when NOD1 agonist Tri-DAP was used. Further, the effect was not observed in murine bone marrow derived neutrophils, potentially because of impurity of the thioglycollate-elicited peritoneal exsudate neutrophils. IL-1β secretion of murine bone marrow derived neutrophils was NALP3 (NLRP3)-dependent after combined stimulation with LPS and nigericin (Mankan et al., 2011). Therefore, the control stimulus used here might have been insufficient, although MDP has been described to activate the NALP3 inflammasome (Netea et al., 2010). Here, the production of MIP-1α, RANTES and TNF was unimpaired in NOD1-, NOD2- and NALP3-deficient Hoxb8 neutrophils after *A. phagocytophilum* infection. This means that these NLR are probably not involved in the recognition of *A. phagocytophilum* by neutrophils. However, the respective pathways might not be active in Hoxb8 neutrophils because a chemokine and cytokine response after stimulation with NOD agonists was not detected.

*In vitro*, growth of *A. phagocytophilum* in NOD1^-/-^, NOD2^-/-^ and NALP3^-/-^ Hoxb8 neutrophils was not better than in WT control cells. However *in vivo*, NOD2^-/-^ mice had elevated bacterial loads in the early phase of infection compared to WT animals. This finding has to be interpreted with caution because the NOD2-deficient mice were on a C57BL/6J background in contrast to the C57BL/6N control animals. However, it is in line with the previous finding that mice deficient for RIP2 (RIPK2), the adaptor molecule of the NOD1 and NOD2, showed delayed clearance of *A. phagocytophilum* (Sukumaran et al., 2012). Therefore, it could be that other cell types than neutrophils recognize *A. phagocytophilum via* NOD2. In contrast, the course of infection in NALP3^-/-^ animals did not differ significantly from WT mice (Pedra et al., 2007a).

### CLR-Dependent Recognition of *A. phagocytophilum*

Murine neutrophils express various CLR (Thomas and Schroder, 2013). CLR pathways involve amongst others the adaptor/signaling proteins DAP12, FcRγ and SYK (Brown et al., 2018). Ligand binding leads to production of reactive oxygen species and cytokine secretion (Thomas and Schroder, 2013). To our knowledge, no data exist whether CLR stimulation induces iNOS in neutrophils. However, iNOS mRNA and NO production were induced in murine peritoneal macrophages by the CLR CD23 (FcϵRII) *via* FcRγ-dependent signaling after α-mannan-stimulation (Guo et al., 2018). Here, HKMT induced iNOS mRNA in WT, DAP12^-/-^ and FcRγ^-/-^ Hoxb8 neutrophils. At least in FcRγ^-/-^ Hoxb8 neutrophils, an effect on iNOS induction could have been expected (Geijtenbeek and Gringhuis, 2009), but it might have been masked by TLR2-stimulation through HKMT (Underhill et al., 1999).

In essence, the amount of MIP-1α, RANTES, TNF and IL-6 in the supernatants of DAP12^-/-^ and FcRγ^-/-^ Hoxb8 neutrophils was not significantly different to WT control cells after stimulation with HKMT. Other PRR ligands of HKMT might have mitigated an potential effect as discussed above. However, DAP12^-/-^ Hoxb8 neutrophils produced significantly more RANTES and IL-6 than WT cells after HKMT- or LPS-treatment. A similar observation was made earlier for murine DAP12^-/-^ bone marrow derived DC (Chu et al., 2008) and murine bone marrow derived DAP12^-/-^ macrophages (Hamerman et al., 2005) that was interpreted as negative regulation of the TLR-response by DAP12. Here, we show that this is also true for Hoxb8 neutrophils.

iNOS mRNA induction and chemokine and cytokine production after *A. phagocytophilum* infection were unimpaired in DAP12- and FcRγ-deficient Hoxb8 neutrophils. Further, bacterial growth in Hoxb8 neutrophils was not significantly better than in WT control cells. *In vivo*, bacterial loads in DAP12^-/-^ mice, FcRγ^-/-^ mice and mice with myeloid cells defective for SYK were able to control *A. phagocytophilum* as efficiently as WT mice. Thus, CLR signaling *via* DAP12, FcRγ, and SYK might not be involved in the recognition of *A. phagocytophilum*. However, the *in vitro* data have to be interpreted with caution because we could not prove whether DAP12- or FcRγ-dependent CLR signaling is active in Hoxb8 neutrophils.

## Conclusion

We showed here that *A. phagocytophilum* is recognized by murine Hoxb8 neutrophils *via* TLR- and MyD88-dependent signaling. However, the TLR involved could not be reliably determined. It seems that the absence of TLR7 or TLR9 alone does not significantly impair stimulation of Hoxb8 neutrophils upon *A. phagocytophilum* infection. Probably, more than one TLR is involved. *In vivo*, the absence of MyD88 seems to be compensated because of the redundancy of the immune system. Hoxb8 neutrophils defective of the NLR and CLR pathways investigated were unimpaired in recognition of *A. phagocytophilum*. In other cell types than neutrophils NOD2 might contribute to immune stimulation, because *in vivo* NOD2 mice showed delayed clearance of *A. phagocytophilum* in the early phase of infection.

## Data Availability Statement

The original contributions presented in the study are included in the article/**Supplementary Material**. Further inquiries can be directed to the corresponding author.

## Ethics Statement

The animal study was reviewed and approved by the animal welfare committee of the Regierungspräsidium Freiburg (G-06/19 and G-11/79).

## Author Contributions

Substantial contributions to the conception: BM, AW, and FL. Design of the work: FL. Acquisition of data: BM, AW, KB, BS, SK, and FL. Analysis of data: BM, AW, and FL. Interpretation of data: BM, AW, and FL. Drafting the work: FL. Revising it critically for important intellectual content: BM, AW, CB, and FL. Final approval of the version to be published: BM, AW, KB, BS, SK, CB, and FL. Agreement to be accountable for all aspects of the work in ensuring that questions related to the accuracy or integrity of any part of the work are appropriately investigated and resolved: BM, AW, KB, BS, SK, CB, and FL. All authors contributed to the article and approved the submitted version.

## Funding

This research was supported by the German Research Foundation (grant LO 1163/1-2).

## Conflict of Interest

The authors declare that the research was conducted in the absence of any commercial or financial relationships that could be construed as a potential conflict of interest.

## Abbreviations

CLR, C-type lectin receptor; FcRγ, Fc-receptor γ-chain; HKMT, heat-killed mycobacteria; iNOS, inducible nitric oxide synthase; NLR, Nod-like receptor; PRR, pattern recognition receptor; p.i, post infection; TLR, Toll-like receptor; WT, wild-type.
